# Treatment of Regional Metastatic Melanoma of Unknown Primary Origin

**DOI:** 10.3390/cancers7030849

**Published:** 2015-08-10

**Authors:** Elke J. A. H. van Beek, Alfons J. M. Balm, Omgo E. Nieweg, Olga Hamming-Vrieze, Peter J. F. M. Lohuis, W. Martin C. Klop

**Affiliations:** 1 Department of Head and Neck Surgery and Oncology, The Netherlands Cancer Institute-Antoni van Leeuwenhoek, Plesmanlaan 121, 1066CX, Amsterdam, The Netherlands; E-Mails: el.v.beek@nki.nl (E.J.A.H.B.); a.balm@nki.nl (A.J.M.B.); p.lohuis@nki.nl (P.J.F.M.L.); 2 Melanoma Institute Australia, 40 Rocklands Rd, North Sydney NSW 2060, Australia; E-Mail: Omgo.Nieweg@melanoma.org.au; 3 Department of Radiotherapy, The Netherlands Cancer Institute-Antoni van Leeuwenhoek, Plesmanlaan 121, 1066CX, Amsterdam, The Netherlands; E-Mail: o.vrieze@nki.nl; 4 Skin and Melanoma Centre, The Netherlands Cancer Institute-Antoni van Leeuwenhoek, Plesmanlaan 121, 1066CX, Amsterdam, The Netherlands

**Keywords:** melanoma, unknown primary, surgical treatment

## Abstract

(1) Background: The purpose of this retrospective study was to evaluate the recurrence and survival rates of metastatic melanoma of unknown primary origin (MUP), in order to further refine current recommendations for the surgical treatment; (2) Methods: Medical data of all MUP patients registered between 2000 and 2011, were analyzed. Seventy-eight patients were categorized in either lymph node (axilla, groin, head-and neck) or subcutaneous MUP. Axillary node MUPs were generally treated with dissections of levels I-III, inguinal node MUPs with combined superficial and deep groin dissections, and head-and-neck node MUPs with neck dissections to various extents, based on lymph drainage patterns. Subcutaneous lesions were excised with 1–2 cm margins. The primary outcome was treatment outcomes in terms of (loco)regional recurrence and survival rates; (3) Results: Lymph node MUP recurred regionally in 11% of patients, with an overall recurrence rate of 45%. In contrast, subcutaneous MUP recurred locally in 65% of patients with an overall recurrence rate of 78%. This latter group had a significantly shorter disease-free interval than patients with lymph node MUP (*p* = 0.000). In the entire study population, 5-year and 10-year overall survival rates were 56% and 47% respectively, with no differences observed between the various subgroups; (4) Conclusion: The relatively low regional recurrence rate after regional lymph node dissection (11%) supports its current status as standard surgical treatment for lymph node MUP. Subcutaneous MUP, on the contrary, appears to recur both locally (65%) and overall (78%) at a significantly higher rate, suggesting a different biological behavior. However, wide local excision remains the best available option for this specific group.

## 1. Introduction

In approximately 3% of all melanoma cases, patients present metastatic melanoma in either lymph nodes, (sub)cutaneous tissue, or visceral sites, without a detectable primary tumor, despite extensive examinations [1]. The apparent absence of a primary tumor is not yet fully understood. One theory holds that immune surveillance may have caused the primary malignancy to regress at an early stage of its development [2,3]. Alternatively, there may have been a prior lesion at the site that at the time had been excised without proper histologic analysis, or ectopic nodal nevus cells may have turned malignant in regional lymph nodes [4,5]. 

The most common clinical presentation of metastatic melanoma of unknown primary origin (MUP) is lymph node disease without clinical or radiological evidence for visceral involvement [6]. As the primary site is unknown, little can be said about the regional or distant nature of solitary lymph node metastases, but they commonly develop in axilla (54%), neck (26%), and groin (20%) [4,7].

In 12%–39% of MUP patients, the disease manifests in subcutaneous tissue [4,8,9]. For such solitary lesions, it is even more difficult to differentiate between a primary cancer, regional and distant metastatic disease, as these lesions may as well represent a primary melanoma without epidermal components, a lymphogenic in-transit metastasis, or even a hematogenous metastasis [10,11]. 

Literature suggests that both lymph node and subcutaneous MUP have a better prognosis than stage III melanoma with a known primary site [7,12]. Therefore, the American Joint Committee on Cancer (AJCC) recommends that MUP be classified as stage III, rather than stage IV disease, thus assuming the MUP lesions to be manifestations of regional disease [13].

In spite of the absence of level 1 and 2 evidence, surgical treatment with curative intent is the standard of care for MUP. For lymph node MUP, lymph node dissection is recommended, and for (sub)cutaneous lesions the standard of care is wide local excision with minimal margins of 1 cm [10]. 

Our tertiary referral cancer center, follows the AJCC recommendations for MUP, and therefore we generally perform (loco-) regional surgery for this disease. The purpose of this study was to evaluate the recurrence and survival rates of MUP, to further refine the current treatment guidelines.

## 2. Results

Between January 2000 and December 2011, 3491 patients with melanoma were treated in our institute, of whom 155 (4%) were registered with MUP. After thorough analysis, 77 patients were excluded for the following reasons. Twenty patients appeared to have proven or suspected primary melanomas; 52 already had visceral metastases at first presentation; one patient had a metastatic lesion in an unusual basin (epitrochlear); three patients lacked sufficient data to confirm the diagnosis of MUP; and one patient on immunohistochemical evaluation had turned out to have a sarcoma. The remaining 78 patients with histologically or cytologically proven MUP were enrolled.

### 2.1. Patient Characteristics 

Median follow-up for the entire population was four years (range 0.5–17 years). Only one patient was lost to follow-up at seven months after treatment. Table 1 presents patient characteristics and subgroup distributions. There were no significant differences between the subgroups in terms of sex and age. Seven patients had prior skin lesions that had been removed but not histologically investigated, or had regressed spontaneously. 

**Table 1:** Baseline patient characteristics.

	N	%	Details
**Gender**			
Male	46	59	
Female	32	41	
**Age**			
Median	55		
Range	15–72		
**Subgroups**			
Lymph node	55	71	
*Axilla*	*27*	*35*	
*Head and Neck*	*18*	*23*	
*Groin*	*10*	*13*	
Subcutaneous	23	29	6 with nodal metastasis: Subcutaneous lesion scapula R and axilla L ^a^ Subcutaneous lesion lateral malleolus L and groin L Subcutaneous lesion thigh L and groin L Subcutaneous lesion retroauricular region L and level II R Subcutaneous lesion supraclavicular fossa R and level V R Subcutaneous lesion lower eyelid L and parotid gland L

^a^ L: left, R: right.

In 29 patients (37%), the initial operation was performed in referring hospitals, including 13 axillary lymph node dissections, two neck dissections, one therapeutic excision of a suboccipital node, three inguinal lymph node dissections, and 10 therapeutic excisions of subcutaneous lesions. Three patients underwent additional surgery to meet our own surgical guidelines. 

### 2.2. Treatment Regimens

In 22 patients with axillary node disease, a standard lymphadenectomy of levels I–III was performed, one patient had a level I and II dissection, and four had dissections of unknown extent. In the head and neck subgroup, four patients underwent neck dissections of a different extent than the proposed standard. One of them had a metastasis at level V and underwent a level II–V dissection combined with a subnuchal node dissection. Another patient had a parotidectomy combined with selective neck dissection of levels II and III for a parotid gland metastasis. A third patient had a level II metastasis, for which a neck dissection with additional superficial parotidectomy was performed. In a fourth patient a suboccipital metastasis had been excised without adjuvant neck dissection. 

Nine patients with inguinal lymph node disease were treated in the standard fashion. For one patient, the exact data on the extent of surgery was missing. In the (sub)cutaneous subgroup, six patients with both subcutaneous lesions and lymph node involvement underwent lymphadenectomies according to protocol. 

In seventeen patients (74%) in the (sub)cutaneous group, the wide excision margins were histologically negative, in three patients (13%) positive and in three patients (13%) this data was unknown. 

Fifteen patients (56%) with axillary involvement, 11 patients (61%) in the head and neck subgroup, two patients (20%) with inguinal metastases and five patients (22%) with (sub)cutaneous lesions had received adjuvant radiation therapy. 

### 2.3. Recurrences

Table 2 lists the treatment results and recurrence figures during follow-up, specified as locoregional, systemic, or both. Forty-three patients recurred, accounting for a 45% recurrence rate in the lymph node group and a 78% recurrence rate in patients with (sub)cutaneous metastases. Overall, patients with lymph node involvement had a significantly longer DFI than those with (sub)cutaneous metastases, with medians of 8.7 and 0.6 years, respectively. Recurrences in the dissected lymph node basin developed in 15% of the axilla subgroup, 6% of the head and neck subgroup and 10% of the groin subgroup, adding up to a total regional recurrence rate of 11% in patients with lymph node metastases. The local recurrence rate was 65% in patients with (sub)cutaneous lesions.

**Table 2:** Recurrence of disease per subgroup. Locoregional (LR) and/or systemic (Sys) recurrence of disease per subgroup, in patients receiving surgical treatment according to (+) or not according to (−) the guidelines.

MUP ^a^	N (%)	Initial Surgical Treatment ^b^			Location Recurrence ^c^			Overall Disease-Free Interval (years)				
		+	−	Unknown	LR	Sys	Both	Median	1 year	2 year	5 year	
**S**	18/23 (78)	18	0	0	7	3	8	0.6	30	22	17	*P* = 0.000
**LN**	25/55 (45)	16	4	5	1	19	5	8.7	69	65	56	
**A**	15/27 (56)	10	1	4	0	11	4					
**HN ^d^**	6/18 (33)	3	3	0	1	5	0					
**G**	4/10 (40)	3	0	1	0	3	1					

^a^ MUP: metastatic melanoma of unknown primary origin (S: subcutaneous, LN: lymph node, HN: head&neck, A: axilla, G: groin), ^b^ + standard treatment, − non-standard treatment, ^c^ LR: locoregional, Sys: systemic, Both: locoregional and systemic, ^d^ 1 lost to follow-up.

### 2.4. Survival 

**Figure 1:**
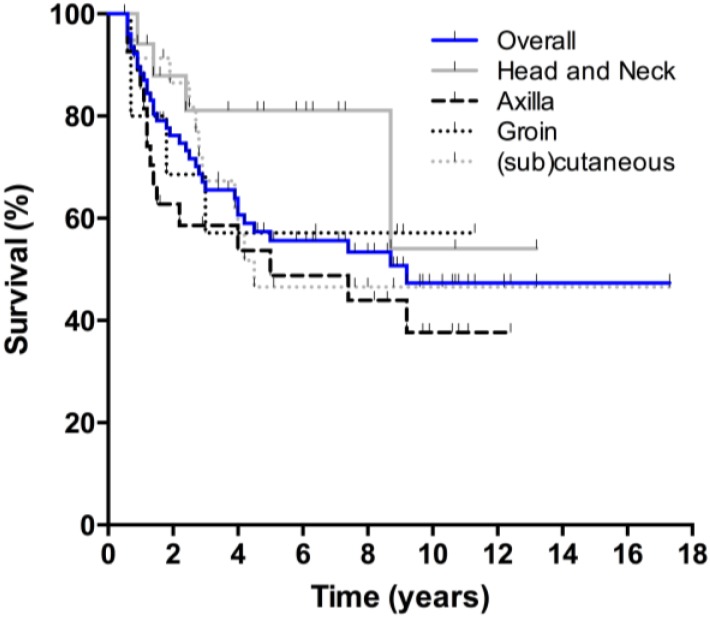
Overall survival. Overall survival of the total study population (blue), and for each subgroup; head and neck, axilla, groin, and (sub)cutaneous.

**Figure 2:**
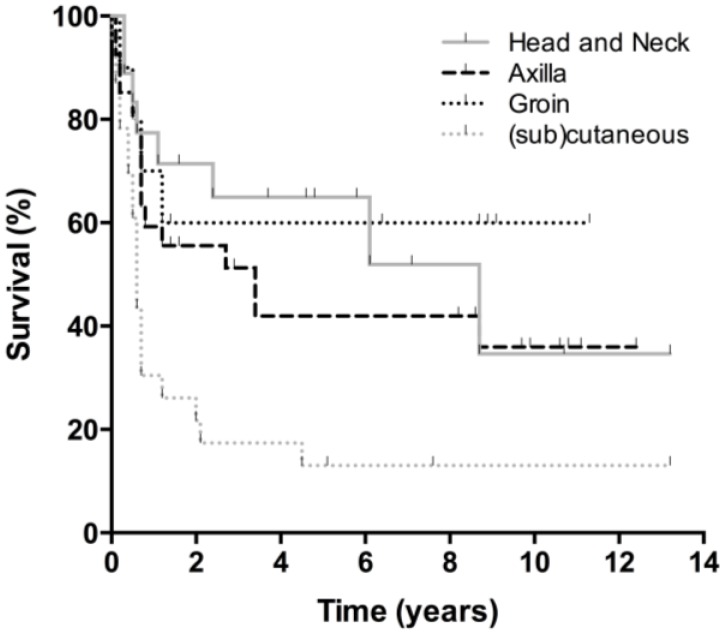
Disease-free survival per subgroup. Disease-free survival for each subgroup; head and neck, axilla, groin, and (sub)cutaneous.

**Figure 3:**
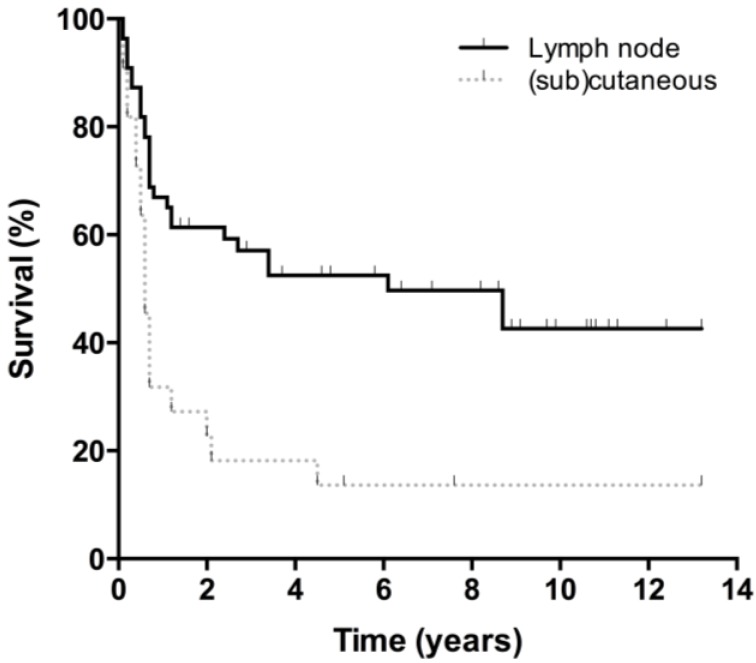
Disease-free survival lymph node *vs.* (sub)cutaneous MUP. Disease-free survival for patients with lymph node MUP and (sub)cutaneous lesions.

Overall survival curves are presented in Figure 1. Thirty-four patients died during follow-up, 25 from melanoma, seven from an unrelated cause, and two from an unknown cause. Five-year and 10-year OS were 56% and 47% respectively, with no differences between men and women. The lymph node subgroup had a 5-year and 10-year OS of 60% and 47%, respectively. Twenty-three patients in this subgroup (42%) were followed beyond five years. After ten years, 38% of patients with axillary involvement were still alive, compared to 54% of the head and neck subgroup and 57% of the groin subgroup (ns). All patients with (sub)cutaneous metastases who died had done so within five years after treatment, accounting for a 5-year and 10-year survival of 47%. No significant differences in OS were observed between the lymph node and (sub)cutaneous group. DFS was significantly better for patients with lymph node involvement than for those with (sub)cutaneous lesions (Figure 2 and Figure 3). Disease specific survival was similar in both groups.

### 2.5. Subgroup Analysis

In terms of survival and recurrence, no differences were observed between patients with or without adjuvant radiotherapy. In patients with both lymph node and (sub)cutaneous metastases, however, OS appeared slightly worse compared to those with only one of these entities (*p* = 0.072).

## 3. Discussion

This study demonstrates a relatively low regional recurrence rate (11%) after regional lymph node dissection with or without postoperative radiotherapy, supporting our current regional treatment protocol in MUP patients. 

Eighty-one percent of patients with axillary involvement had undergone dissection of levels I, II and III. In this subgroup, 56% recurred, with four cases of regional recurrence. Five-year OS was 49%, which is in line with other studies [6,14]. These results indicate that our surgical guidelines for axillary lymph node MUP are adequate and no modifications are needed. In the head and neck subgroup, only one patient recurred regionally, and the overall recurrence rate was 33%. Five-year survival was 81%, which is high compared to the 39%–44% reported in other studies [14,15]. These results indicate that the treatment regimens in this subgroup, too, were adequate. We assume a lymph node MUP in the neck or parotid gland to be a metastasis from a hypothetical primary (completely regressed) tumor in the head and neck region. Thus, the probable location of this primary tumor can be located by retracing the lymph drainage pattern. Lymph drainage patterns of melanoma in the head and neck have been described before, with tumors anterior to the so-called watershed line usually draining to the parotid gland and levels I–V, and posterior tumors usually draining towards the suboccipital region and levels II–V [16,17]. The guidelines in our institute for neck dissections in MUP are based on these predictable drainage pathways. Ninety percent of patients with an inguinal metastasis underwent a combined superficial and deep groin dissection. The disease recurred in 40% of these patients, with only one case of regional recurrence. Five-year OS was high with 57% *versus* the 35%–47% range reported in literature, again confirming a safe surgical approach for this particular type of MUP [6,14]. 

Patients with (sub)cutaneous MUP had a high overall recurrence rate of 78%, with a significantly shorter DFI (0.6 *versus* 8.7 years) compared to patients with lymph node metastases. The 5-year OS of 47% in the (sub)cutaneous subgroup remains within the range of 25%–73% reported in other studies [4,8,10]. DFS in this group was significantly lower than in the lymph node group. To our knowledge, no other studies have reported on the differences in terms of recurrence rate and DFS between lymph node and (sub)cutaneous MUP. These divergent outcomes may indicate a difference in biological nature between the two entities, possibly related to a distant metastatic nature of subcutaneous lesions. Admittedly, OS and DSS in both conditions are similar, due to the equal number of deaths of diseases. Yet the significant discrepancy in recurrence rates is a clear sign of a more aggressive disease in (sub)cutaneous MUP. Differences in survival may have simply remained concealed because of our underpowered study population. Adding to this point, OS after locoregional treatment appeared slightly lower (*p* = 0.072) in a small subgroup analysis of six patients with both lymph node and subcutaneous metastases. However, Jonk and colleagues (1994) did not demonstrate a worse prognosis for this group [18]. 

In view of these outcomes adjuvant immunotherapy may be indicated, though is not yet available. 

In the current study, MUP patients with or without adjuvant radiotherapy did not differ in recurrence and survival rates despite the presence of adverse prognostic factors in the irradiated patients (data not shown). Several retrospective series on the role of adjuvant irradiation in the management of metastatic melanoma to regional lymphatic basins demonstrated an improved regional control after adjuvant radiotherapy in all subsites in patients with risk factors such as multiple involved lymph nodes, extracapsular extension, large nodes or recurrent disease [19,20,21]. Recently, a randomised trial from the Trans-Tasman Radiation Oncology Group (TROG) was published confirming that adjuvant radiotherapy improves lymph node field control in patients at high risk of regional relapse after lymphadenectomy, but no differences were noted for relapse-free survival or overall survival [22]. Based on these results, adjuvant radiotherapy should be discussed with patients at high risk of relapse after lympadenectomy, to prevent the morbidity of a regional recurrence including pain, ulceration, malodour, lymphoedema, and impaired function. On the other hand, the disadvantages of adjuvant radiation therapy need to be taken into account. Besides possible morbidity such as oedema and fibrosis, these could include a more difficult identification of recurrences, or hamper salvage surgery.

We have to emphasize the limitations of a retrospective study design on our patient and treatment selection. Despite a potential selection bias on account of our tertiary referral position, the 4% incidence of MUP in our melanoma population was within the range of percentages (2%–6%) reported in the literature [4,23,24]. Also in line with international reports were other epidemiological features, like a median age of 55 years and a male preponderance of 59% [6,10,25]. Why male patients are frequently overrepresented, remains unclear [10,14]. The distribution of lymph node and (sub)cutaneous metastatic disease that we found, also concurs with data reported by others, with predominant locations in the axilla and on the extremities [10,23,26]. Seven patients turned out to have had prior skin lesions which had been removed without proper histologic analysis or which had regressed spontaneously. In theory, their excised lesions could well have been primary melanomas. As for the lesions that had spontaneously regressed, there may, of course, have been a lot more in other patients, too, which may simply have gone unnoticed. All the same, these seven patients were not excluded, as they did meet the international criteria for MUP. No significant differences in survival have been reported between patients with and without histories of prior regressed skin lesions [18].

In conclusion, the findings concerning lymph node MUP in terms of recurrence and survival compare favorably to international literature and therefore support the existing surgical guidelines. For neck and groin lymph node MUP, more data are needed to reach consensus on dissection guidelines. 

Recurrence and DFS in (sub)cutaneous MUP differ so much from the figures for lymph node MUP, that solitary subcutaneous metastases perhaps should be considered a different disease altogether showing different biological behavior. 

## 4. Materials and Methods

Medical data were analyzed of all MUP patients registered at our institute between January 2000 and December 2011 with clinical and pathologic evidence of melanoma limited to a lymph node basin or (sub)cutaneous tissue, without epidermal components. In all patients, no suspected primary source had been found despite thorough examination, including full-body inspection of the skin. Patients with multiple subcutaneous metastases were included only if the lesions were located in the same lymphatic drainage region. In case of both lymph node and subcutaneous metastases, the lesions had to be located in the same anatomic area. Patients with evident stage IV disease on first presentation were excluded. 

Data on patient demographics, disease characteristics, extent of surgery, local or distant nature of recurrences, and (disease-specific) survival was retrieved from the medical records. If the medical records were incomplete, GPs and referring medical specialists were consulted. Follow-up started after the operation and ended by definition in April 2013. The patients who were alive by then were censored. 

The study population was categorized according to metastatic site into (sub)cutaneous MUP and lymph node MUP. The latter group was subdivided into axillary, head and neck, and groin MUP. Patients with both lymph node and skin involvement were ranked among the (sub)cutaneous subgroup, based on the prognosis. Patients with metastatic disease in multiple lymph nodes basins were ranked according to the basin where most pathologically confirmed lymph node metastases had been found.

### 4.1. Treatment Regimens 

All MUP patients received surgical treatment according to the following guidelines. Axillary lymph node disease was treated with dissection of levels I–III. For neck metastasis a dissection of levels I–V or II–V was performed, depending on the particular lymph drainage patterns as described by O’Brien *et al*. [16]. Parotid lymph node metastases were removed by parotidectomy with en bloc comprehensive neck dissection. Suboccipital nodes were removed with neck dissection of levels II–V and subnuchal node dissection. Inguinal metastatic lymph nodes were treated with combined superficial and deep (iliac and obturator) groin dissection. (Sub)cutaneous lesions, finally, were removed by wide local excision with 1–2 cm margins.

Post-operative radiotherapy was indicated to improve regional control in patients with multiple involved neck metastases and, in case of axillary and groin metastasis, in patients with tumor positive resection margins, gross extracapsular spread or massive tumor involvement [19].

### 4.2. Statistical Analysis

Disease-free interval (DFI), overall survival (OS), disease-free survival (DFS), and disease-specific survival (DSS) were calculated with the Kaplan-Meier method. DFI was calculated from the date of the operation until the first recurrence, and OS and DFS from the date of the operation to death. Survival among the various subgroups was compared using the log rank test. SPSS statistical software was used for all analyses. A *p* value < 0.05 was considered statistically significant.
